# Left ventricular lead delivery system used to implant right ventricular lead via persistent left superior vena cava

**DOI:** 10.1002/joa3.12823

**Published:** 2023-02-02

**Authors:** Bekir Serhat Yildiz, Ramazan Gündüz, Su Ozgur

**Affiliations:** ^1^ Department of Cardiology Celal Bayar University School of Medicine, Hafsa Sultan Hospital Manisa Turkey; ^2^ Department of Cardiology Manisa City Hospital Manisa Turkey; ^3^ Department of Biostatistics Ege University School of Medicine Izmir Turkey

**Keywords:** coronary sinus sheath, pacemaker implantation, persistent left superior vena cava

Persistent left superior vena cava (PLSVC) is a congenital anomaly of thoracic venous system and is observed in 0.3%–0.5% of general population. Persistent left superior vena cava can be diagnosed as an isolated form or associated with other right superior vena cava abnormalities.^1^, ^2^ The diagnosis of persistent left superior vena cava could be conducted incidentally during the implantation process of pacemaker (PM) or central venous catheter. Thus, it should be emphasized that the techniques for PM implantation through the PLSVC are quite rare.^3^, ^4^, ^5^

In this case, we have elaborated on the successful and rapid implementation of PM implantation and right ventricular (RV) lead placement in tortuous persistent left superior vena cava via coronary sinus (CS) sheath technique.

A 90‐year‐old patient was referred to a different cardiology institution due to syncope and total atrioventricular (AV) block. A temporary pacemaker had been implanted by the right femoral approach. Ejection fraction had been calculated as 58% on echocardiography by the Simpson method. An enlarged CS was observed after injection of contrast bubbles by the left arm on transthoracic echocardiography, confirming persistent left superior vena.

One day later, it was planned to perform a ventricular demand pacing (VVI) on the patient. The medical team tried to access the right subclavian vein, but the guidewire did not reach the RV zone. The venography denoted that right vena cava superior was drained to different vein (Video 1). At this stage, they then decided to utilize left subclavian vein for pacemaker implantation but detected a tortuous persistent left superior vena (Video 2). They tried to access RV through persistent left superior vena and CS via catheter delivery sheath (Model C315–S10, Medtronic), but failed to achieve VVI pacemaker implantation due to tortuous PLSVC. Consequently, the procedure was cancelled after several attempts.

Venography of right superior vena cava. Right superior vena cava drains into right renal vein. To view this video in the full‐text HTML version of the article, please visit https://onlinelibrary.wiley.com/doi/10.1002/joa3.12823.

Venography of left superior vena cava. A tortuous and acute angle of left superior vena cava. To view this video in the full‐text HTML version of the article, please visit https://onlinelibrary.wiley.com/doi/10.1002/joa3.12823.

This patient was referred to our institution with a temporary PM. We have performed a computerized tomography (CT), confirmed PSLVC and draining of the right superior vena cava to the right renal vein and schematic anatomy of upper venous system was illustrated (Figure 1). We decided to place VVI permanent pacemaker on the left side of the patient. The left subclavian vein had been punctured and a 7 French (F) sheath was implanted. A right diagnostic coronary angiography catheter (Expo™, Femoral right 4, Boston Scientific) has been located to RV apex with the support of hydrophilic wire (REPA, Hydrophilic Guidewire‐180 cm, APT Medical Inc.) passing through tricuspid valve and then the hydrophilic wire has been changed with a super stiff wire (Amplatz Super Stiff ™ Guidewire‐180 cm, Boston Scientific) in right catheter. Right coronary artery catheter and 7F sheath were removed. CS sheath has been placed to RV apex by using super stiff wire and then super stiff wire was removed. VVI ventricular PM lead was located to RV using CS sheath [Attain Command™ + SureValve™ 6250 V‐EH (Extended Hook), Medtronic] (Figure 2). We have successfully positioned the CS sheath‐implanted RV lead with active fixation through the PLSVC to the apex proportion of RV. The sensing and capture thresholds were acceptable. The sheath was successfully removed by cutting and the lead was connected to a PM generator (Video 3). The full procedure took 25 and 6 min was belong to fluoroscopy procedures.

**FIGURE 1:**
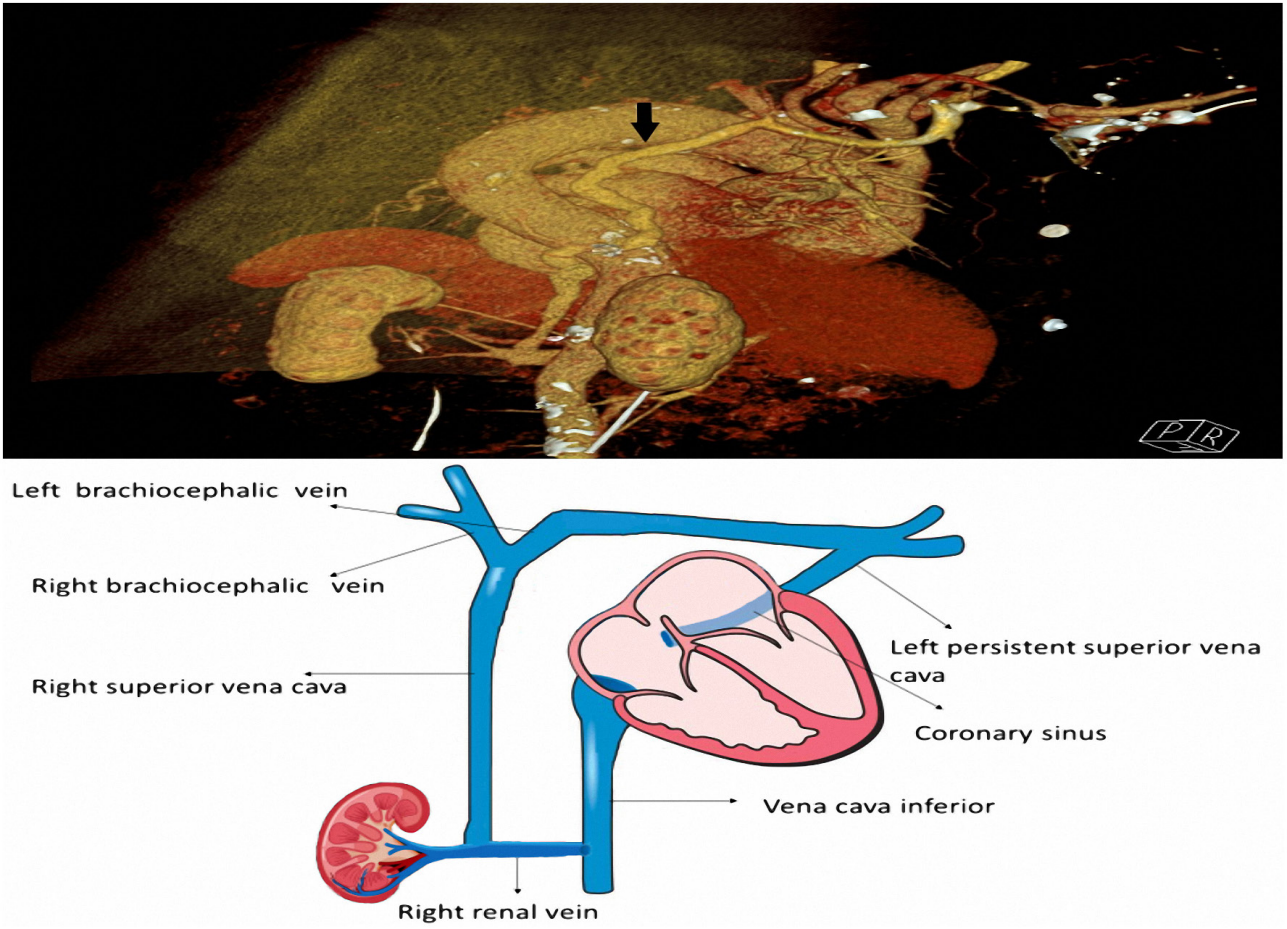
(A) Computerized tomography of thoracic venous system (upper figure). (B) Schematic anatomy of upper venous system (below). Persistent left superior vena cava drains into coronary sinus and right superior vena cava (black arrow) drains into right renal vein.

**FIGURE 2:**
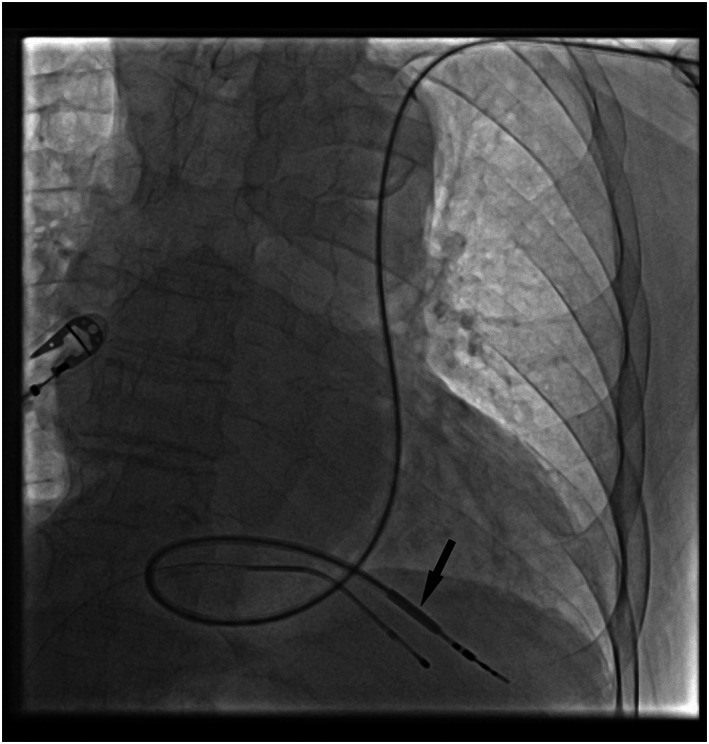
X‐ray imaging of implantation of right ventricular lead. Right ventricular lead was located into right ventricle through coronary sinus sheath (black arrow).

X ray of chest after pacemaker implantation. To view this video in the full‐text HTML version of the article, please visit https://onlinelibrary.wiley.com/doi/10.1002/joa3.12823.

In this case, we have elaborated an uncommon case of a tortuous PLSVC in whom we implanted RV lead using a CS sheath technique. Although Attain Command guiding catheter series have various forms and sizes of catheters we choose ‘Attain Command™+SureValve™ 6250V‐EH, Medtronic’. This catheter length is 50 cm. It is very suitable for moving in tortuous veins. By turning this sheath to the counterclockwise, its tip can turn toward the tricuspid orifice, advance across the tricuspid valve easily due to distal curve. Also, this sheath was located easily into right ventricle and made good stabilization at right ventricle apex after we removed Amplatz super stiff wire. We especially choose this catheter due to distal curved shape, proximal wide angle and enhanced torque control. To our knowledge, it is the first time that this technique has been used in the placement of PM lead via PLSVC in literature. Some other techniques were used for implantation of PM in different cases who had PLSVC like anchor balloon, C type stylet or catheter delivery system.^3^, ^4^, ^5^

The abnormal anatomy of PLSVC may cause difficulties in lead fixation due to an angle between the orifice of the CS and the tricuspid valve.^3^, ^4^, ^5^ According to this technique, it is easy to pass from PLSVC to RV via tricuspid valve with wire and deliver ventricular PM lead to RV CS sheath in tortuous and calcific vessel with or without PLSVC. In our case passing from tortuous PLSVC with CS sheath has been conducted safely and rapidly without any complications. Other techniques like epicardial pacemaker via surgery and leadless pacemaker were other options for this patient. We did not choose epicardial pacemaker via surgery due to the high operation risk. Also, we could not use leadless pacemaker due to higher cost.

In conclusion, this technique is a novel procedure that can easily be performed in difficult cases with PLSVC. CS sheath, right Judkins coronary artery catheter, super stiff wire, and hydrophilic wire are easy to obtain in health care facilities.

## CONFLICT OF INTEREST STATEMENT

The authors declare that they have no competing interests.

## ETHICAL DECLARATION

The study complied with the Declaration of Helsinki and informed consent has been obtained from the participant.

## PATIENT CONSENT STATEMENT

The patient consent statement was taken.
